# Ulcer occurrence on adjacent toes and hallux valgus deformity after amputation of the second toe in diabetic patients

**DOI:** 10.1186/s13018-023-03577-z

**Published:** 2023-02-13

**Authors:** Ines Unterfrauner, Octavian Andronic, Arnd F. Viehöfer, Stephan H. Wirth, Martin C. Berli, Felix W. A. Waibel

**Affiliations:** Orthopedic Department, Balgrist University Hospital, University of Zurich, Zurich, Switzerland

**Keywords:** Amputation, Second toe, Ulceration, Hallux valgus deformity, BMI, Shape first metatarsal head

## Abstract

**Background:**

Amputation of the second toe is associated with destabilization of the first toe. Possible consequences are hallux valgus deformity and subsequent pressure ulcers on the lateral side of the first or on the medial side of the third toe. The aim of this study was to investigate the incidence and possible influencing factors of interdigital ulcer development and hallux valgus deformity after second toe amputation.

**Methods:**

Twenty-four cases of amputation of the second toe between 2004 and 2020 (mean age 68 ± 12 years; 79% males) were included with a mean follow-up of 36 ± 15 months. Ulcer development on the first, third, or fourth toe after amputation, the body mass index (BMI) and the amputation level (toe exarticulation versus transmetatarsal amputation) were recorded. Pre- and postoperative foot radiographs were evaluated for the shape of the first metatarsal head (round, flat, chevron-type), the hallux valgus angle, the first–second intermetatarsal angle, the distal metatarsal articular angle and the hallux valgus interphalangeal angle by two orthopedic surgeons for interobserver reliability.

**Results:**

After amputation of the second toe, the interdigital ulcer rate on the adjacent toes was 50% and the postoperative hallux valgus rate was 71%. Neither the presence of hallux valgus deformity itself (*r* = .19, *p* = .37), nor the BMI (*r* = .09, *p* = .68), the shape of the first metatarsal head (*r* = − .09, *p* = .67), or the amputation level (*r* = .09, *p* = .69) was significantly correlated with ulcer development. The interobserver reliability of radiographic measurements was high, oscillating between 0.978 (*p* = .01) and 0.999 (*p* = .01).

**Conclusions:**

The interdigital ulcer rate on the first or third toe after second toe amputation was 50% and hallux valgus development was high. To date, evidence on influencing factors is lacking and this study could not identify parameters such as the BMI, the shape of the first metatarsal head or the amputation level as risk factors for the development of either hallux valgus deformity or ulcer occurrence after second toe amputation.

*Trial Registration*: BASEC-Nr. 2019-01791

## Introduction

Vascular and neuropathic diseases, diabetes mellitus [28], infections [4], injuries [16], malignancy [19], severe deformities [29], and other irreversible tissue damages necessitate amputations of toes for further damage control [1–3, 12, 14]. Toe amputations are considered minor surgical procedures; however, the re-amputation rate is quoted high in the literature with up to 60% [12, 14] and this might ensue further surgical and medical treatment [5, 30]. Another complication known to occur after toe amputation is the deviation of the adjacent toes because of loss of buttressing and stabilizing effect against excessive abductor forces and resulting imbalance of the flexor and extensor muscles [24, 25]. The second toe presents an essential support for the first toe. Missing integrity of the second metatarsophalangeal joint in case of amputation is suspected to induce lateral deviation of the first toe and hence development of a hallux valgus deformity [24]. Representing the most common pathology of the forefoot in adults [10], the hallux valgus deformity is associated with pain and impaired mobility, and can cause ulcerations on the first toe itself or on the adjacent rays [6, 31]. However, some authors have not found a correlation between hallux valgus and ulcerations [8, 18]. Additionally, body weight has not been associated with hallux valgus deformity [11, 23]. Contrarily, a round shape of the first metatarsal head might be less stable and more prone for hallux progression due to the missing buttressing effect of the articulation itself [22]. Considering this controversy in the available literature and the significant consequences of ulcerations with development of osteomyelitis and further amputations [12], the aim of this study was to investigate the incidence of interdigital ulcerations on the lateral aspect of the first respectively the medial side of the third toe secondary to amputation of the second toe. Furthermore, its association with the development of hallux valgus deformity, due to the missing abutment of the second toe, was evaluated. Moreover, the influence of other parameters, i.e., the body mass index (BMI), the shape of the first metatarsal head, the amputation level, and the preexistence of a toe deformity was investigated. The objective of this study was to identify factors that initiate or enhance hallux valgus development or interdigital ulcerations on the adjacent toes that might influence the postoperative procedure. For instance, supporting devices for the amputated toe could be introduced as a standard therapy after amputation of the second toe to prevent hallux valgus development.

## Materials and methods

Balgrist University Hospital is a tertiary orthopedic surgery referral center with units for "Foot and Ankle Surgery" and “Technical Orthopedics” (treatment of Charcot neuroosteoarthropathy, diabetic feet, foot complications in peripheral artery disease, chronic wounds, amputations as well as prosthetics and orthotics). This retrospective study was approved by the Cantonal Ethics Committee (BASEC-Nr. 2019-01791) and all study participants signed an informed consent.

### Study cohort

All adult patients with amputation of the second toe due to ulcers or osteomyelitis with an available preoperative, weight-bearing, anteroposterior radiograph of the foot between 2004 and April 2020 were retrospectively analyzed. Patients with amputation of the first toe before or after amputation of the second toe, presence of a hallux rigidus prior to amputation, or use of a prosthetic device on the second toe for prevention of lateral drifting of the first toe were excluded. Patients with disorders of the connective tissue (Marfan syndrome, Ehlers-Danlos syndrome) or the neuromuscular system (cerebral palsy, stroke) were also excluded as those patients are known to be prone to develop a hallux valgus deformity due to hypermobility and spasticity [20]. After a minimum of 24 months after amputation, patients underwent clinical and radiological follow-up.

The primary outcome, development of interdigital ulcerations on the lateral side of the first toe respectively on the medial aspect of the third toe, was assessed during regular postoperative follow-up controls. Moreover, demographic data, BMI, and—if applicable—the time interval from surgery to development of ulceration were obtained. Operative information as the day of surgery, the operation side, the indication for surgery, and the amputation level were further extracted from the medical history. All patients were autonomous and ambulatory, and were postoperatively provided with customized orthopedic footwear including bespoke shoes with adjustment to the individual foot shape in general without filling the space between the first and the third toe with a prosthetic device. Patient adherence to orthopedic footwear was ensured in standardized regular follow-up controls in the outpatient clinic setting.

### Radiographic analysis

**Fig. 1:**
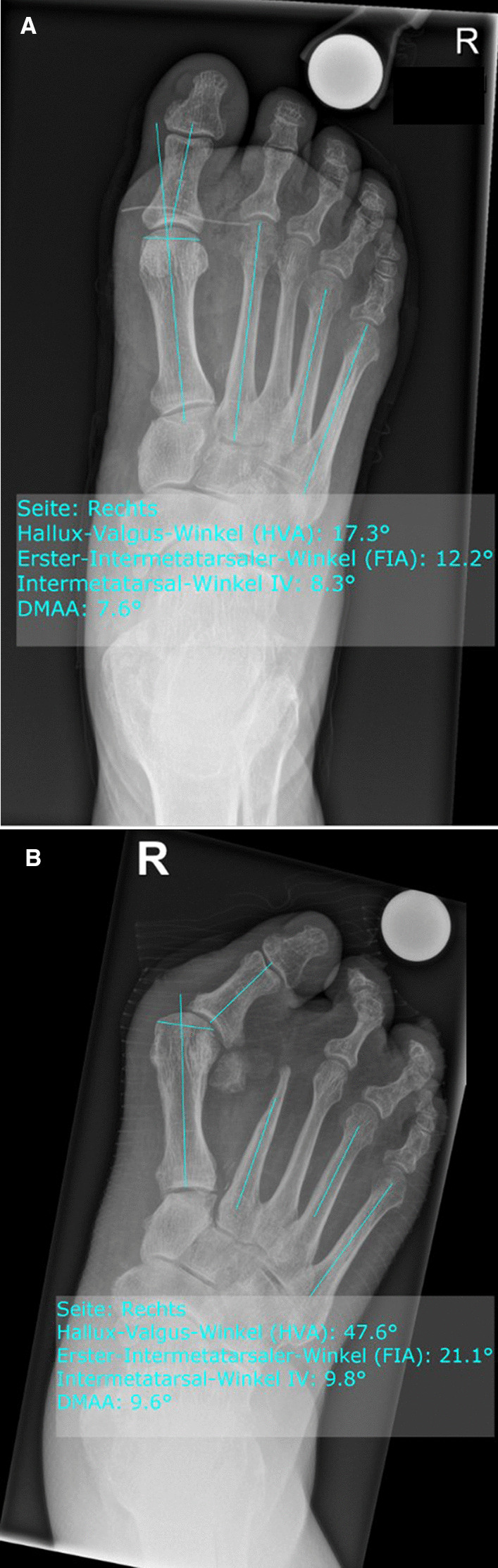
**A**. Preoperative anteroposterior right forefoot radiograph of a 60 years old male patient with marked first metatarsophalangeal angle (HVA), first–second intermetatarsal angle (IMA, here IV) and distal metatarsal articular angle (DMAA). **B**. Postoperative anteroposterior forefoot radiograph of the same patient 5 years after transmetatarsal amputation of the second ray with marked HVA, IMA and DMAA

**Fig. 2:**
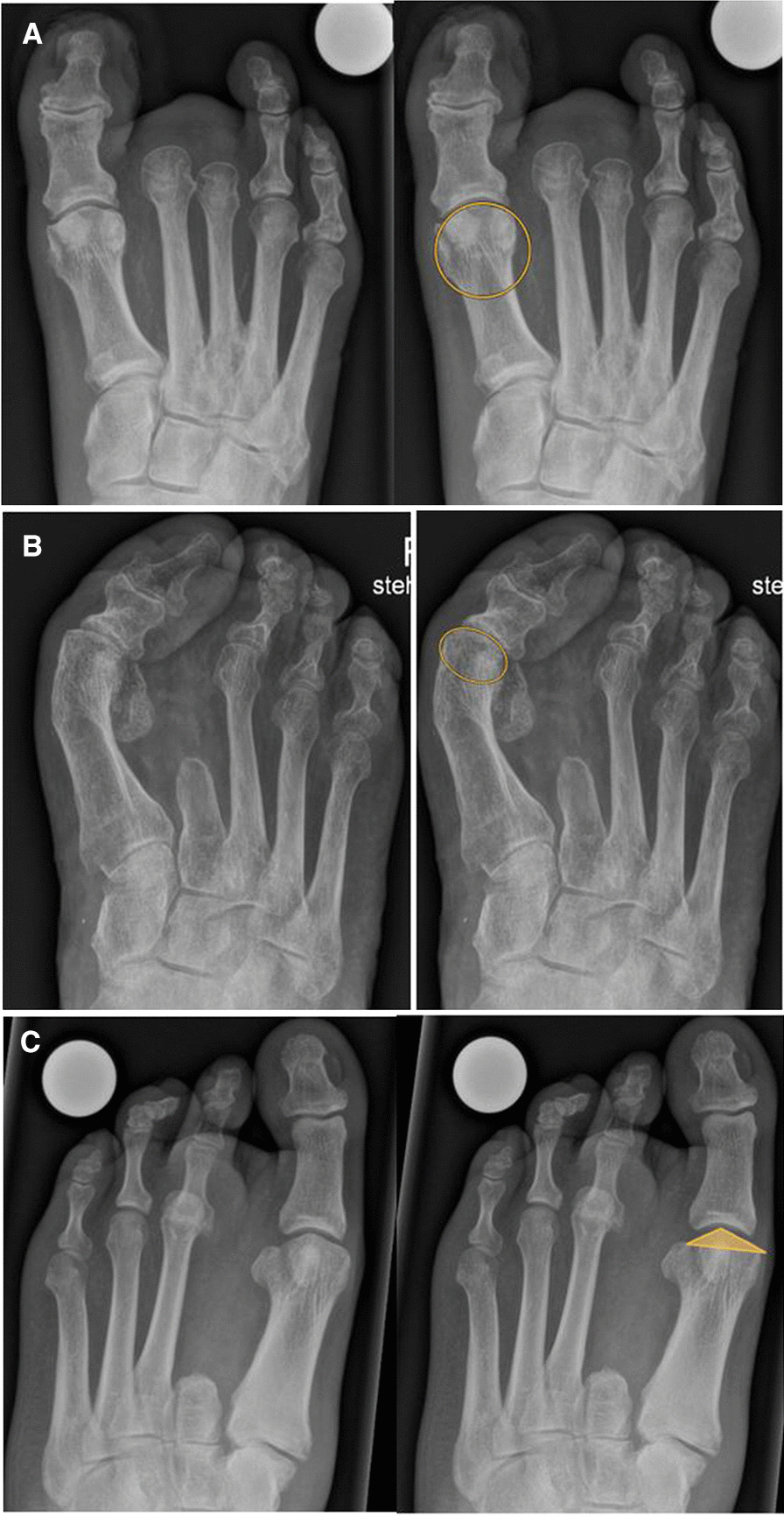
Various types of first metatarsal heads: round (**A**), flat (**B**) and chevron type (**C**)

On the preoperative, weight-bearing, anteroposterior radiograph of the foot, the following parameters were analyzed by two orthopedic surgeons (*IU, OA*): the first metatarsophalangeal angle (known as hallux valgus angle, HVA), the first–second intermetatarsal angle (IMA), the distal metatarsal articular angle (DMAA), the hallux valgus interphalangeal angle (HVI), and the shape of the first metatarsal head (Fig. 1A). Thereby, a distinction between round, flat and chevron type metatarsal head shape was made (Fig. 2). The angular measurements were repeated on the postoperative foot radiograph at least 24 months after amputation (Fig. 1B). The progression of the parameters from the preoperative condition to the position after amputation was calculated. Interobserver reliability was calculated for all measurements.

### Statistical analysis

The primary outcome parameter was the development of interdigital ulcerations at the possible point of mechanical pressure on the first or the third toe after amputation of the second toe. Secondly, the development of hallux valgus and ulcerations on the adjacent toes were correlated. A HVA of ≥ 15 degrees [13], an IMA of ≥ 9 degrees [17], a DMAA of ≥ 10 degrees [17] and a HVI of ≥ 8 degrees [17] were considered pathognomonic for hallux valgus. Further outcome parameters were the association between development of a hallux valgus deformity and the following aspects: the BMI, the shape of the first metatarsal head (flat, round, chevron type), the supporting effect of a totally preserved second metatarsal bone, and the progression of a preexisting hallux valgus deformity. To evaluate the influence of the BMI, patients were divided between those with a BMI higher and lower than 30. The effect of a totally preserved second metatarsal bone was expressed through comparison of patients with and without transmetatarsal amputation. The preexisting hallux valgus deformity prior to amputation was defined according to the preoperative value of the HVA and progression was defined as an increase of the HVA. Groups were compared with the Pearson-Chi^2^-test for categorical variables, and with the Student's *t* and the Mann–Whitney-U-tests for continuous variables. Correlations were carried out with correlation coefficients according to Pearson. IBM SPSS Statistics 25 was used, *p*-values ≤ 0.05 (two-tailed), and correlation coefficients = 0.50 were considered significant respectively strong.

## Results

### Demographic analysis

Twenty-three patients and 24 cases of amputation of the second toe (mean age 68 ± 12 years; 79.2% male) could be included in the study with a mean follow-up of 36 ± 15 months. The mean duration of diabetes mellitus in the study cohort was 21.7 years (± 12.6 years). Table 1 indicated patients' demographic, clinical and treatment characteristics.

**Table 1:** Demographic, clinical and operative characteristics

n = 24	Mean	Standard deviation
Male sex	19 (79.2%)	–
Age at surgery	68 years	12
BMI	30	5.7
Presence of prior hallux valgus	18 (75%)	–
Use of prosthetic device	0 (0%)	–
Round shape of first metatarsal head	17 (70.8%)	–
Ulceration 3rd toe	9 (37.5%)	–
Time interval to amputation	17.9 months	11.9
Ulceration 1st toe	7 (29.2%)	–
Time interval to amputation	21.7 months	11.6
Left operation site	9 (37.5%)	–
Indication for surgery		
Osteomyelitis	21 (87.5%)	–
Deep soft tissue infection	2 (8.3%)	–
Preservation of first metatarsal bone	15 (62.5%)	–

### Interdigital ulcer development and hallux valgus deformity

Overall, twelve cases (50%) showed interdigital ulcerations on the adjacent toes. Five patients showed ulceration on the medial aspect of the third toe, three patients on the lateral side of the first toe and four patients on both, the first and the third toe. From 48 adjacent toes, twelve developed ulcers after amputation of the second toe. Appearance of ulcerations occurred on average 20 ± 11 months after amputation of the second toe.

All radiographic parameters for hallux valgus deformity increased after amputation and all mean postoperative values exceeded physiological values (see lines 118–119) (Table 2a). The HVA increased from 21.3 degrees preoperatively to 26.2 degrees postoperatively, and the IMA from 8.6 degrees to 9 degrees. The progression of HVI was from 17.4 degrees to 20.1 degrees. Although there was a clear trend toward increased hallux valgus deformity in the radiographical assessment, none of the mentioned parameters increased significantly.

**Table 2:** Association of clinical and radiographic parameters with hallux valgus development

a) n = 24	Preoperative	*p* value	Postoperative
HVA (mean)	21.3 (11.9)	0.51^2^	26.2 (21)
IMA (mean)	8.6 (3.2)	0.37^1^	9 (4.3)
DMAA (mean)	9.5 (8.1)	0.90^2^	9.2 (6.5)
HVI (mean)	17.4 (6.4)	0.13^1^	20.1 (8.8)

b) Preexisting hallux valgus deformity; n = 24	Yes	*p* value	No
	n = 18		n = 6
Progression HVA (mean)	5.9 (19.2)	0.54^2^	1.6 (2.8)
Progression IMA (mean)	− 0.8 (4.1)	0.72^2^	1.7 (4.3)
Progression DMAA (mean)	− 1.2 (7.1)	0.82^2^	2.4 (8.9)
Progression HVI (mean)	2.5 (5.6)	0.54^2^	2.9 (2.6)

c) BMI; n = 24	< 30	*p* value	≥ 30
	n = 11		n = 13
Progression HVA (mean)	6.0 (19.4)	1.00^2^	4.0 (14.7)
Progression IMA (mean)	− 0.3 (5.2)	0.25^1^	0.9 (3.3)
Progression DMAA (mean)	1.1 (7.7)	0.21^2^	− 1.5 (8.0)
Progression HVI (mean)	2.2 (5.6)	0.35^1^	3.0 (4.4)

d) Shape first metatarsal head; n = 24	Round	*p* value	Flat or chevron
	n = 17		n = 7
Progression HVA (mean)	7.6 (17.7)	0.11^2^	− 1.7 (12.3)
Progression IMA (mean)	− 0.4 (4.2)	0.17^2^	2.2 (4.0)
Progression DMAA (mean)	0.1 (3.7)	0.36^2^	− 1.2 (14.0)
Progression HVI (mean)	2.8 (5.7)	0.41^2^	2.2 (2.3)

e) Amputation; n = 24	Through MTP	*p* value	Transmetatarsal
	n = 15		n = 9
Progression HVA (mean)	5.2 (16.4)	0.73^2^	4.3 (16.9)
Progression IMA (mean)	− 0.3 (2.7)	0.60^1^	1.5 (5.7)
Progression DMAA (mean)	0 (2.2)	0.82^1^	− 0.7 (12.2)
Progression HVI (mean)	2.9 (5.0)	0.47^1^	2.2 (4.8)

All values are indicated with degree (°), the standard deviation is specified in brackets

Application of Student's *t*-test is indicated with ^1^, application of Wilcoxon rank-sum test (Mann-Whitney U test) is indicated with ^2^

### Associations of clinical and radiographic parameters with ulcerations

#### Preexisting hallux valgus deformity

Eighteen patients (75%) presented with a hallux valgus deformity (HVA ≥ 15 degrees) prior to amputation. Of those, ten (56%) developed an ulceration after amputation of the second toe versus eight (44%) who did not (Table 3). Conversely, also 2 cases without a preexisting hallux valgus deformity developed interdigital ulcerations on the adjacent toes. The difference was not significant (*p* = .35) and there was a weak correlation between preexisting hallux valgus deformity and ulcerations (*r* = 0.19, *p* = .37).

**Table 3:** Association of clinical and radiographic parameters with interdigital ulcerations

n = 24	Ulceration 1st or 3rd toe	No ulceration 1st or 3rd toe	*p* value
Preexisting hallux valgus	10	8	.35
No preexisting hallux valgus	2	4	
Progression HVA (mean)	11	8	.13
No progression HVA (mean)	1	4	
BMI higher 30	8	5	.22
BMI lower 30	4	7	
Round head of first metatarsal	9	8	.65
Flat or chevron type head of first metatarsal	3	4	
Preservation of second metatarsal	8	7	.67
Transmetatarsal amputation	4	5	

Application of Pearson-*χ*^2^ test does not show any significant* p* values (≤ .05)

All values indicate the number of patients

#### Development or progression of hallux valgus deformity

Nineteen cases (79%) either developed a hallux valgus deformity or showed clinical and radiographical signs of progression of a preexisting deformity. Of those, eleven (58%) developed an ulceration versus eight (42%) who did not (Table 3). On the contrary, also one case without radiologic development of a hallux valgus deformity developed an ulceration on the adjacent toes. Again, no significant difference could be found between the groups (*p* = .13) while the correlation between development of hallux valgus deformity and ulcerations was weak to moderate (*r* = .31, *p* = .14).

#### BMI, shape of first metatarsal head, level of amputation

Development of interdigital ulcerations on the adjacent toes was weakly correlated with the BMI (*r* = .09, *p* = .68), weakly negatively correlated with the shape of the first metatarsal head (*r* = − .09, *p* = .67), and again weakly correlated with the amputation level (*r* = .09, *p* = .69) (Table 3).

### Associations of clinical and radiographic parameters with hallux valgus development

#### Preexisting hallux valgus deformity

The 18 cases with a preexisting hallux valgus deformity presented a higher yet not significant progression of the HVA after amputation of the second toe compared to the patients with no hallux valgus deformity prior to surgery (5.9 degrees vs. 1.6 degrees; *p* = .54) (Table 2b).

#### BMI

Thirteen patients of the study cohort had a BMI higher than 30, compared to eleven with a BMI lower than 30. There was no significant difference in the change in radiographic hallux valgus parameters between patients with a higher BMI or lower BMI (Table 2c). Patients with a higher BMI yielded a higher progression of the IMA (0.9 degrees vs. − 0.3 degrees; *p* = .25) and the HVI (3 degrees vs. 2.2 degrees; *p* = .35) compared to the patients with a lower BMI. However, they also showed a not significant lower progression of the HVA (4 degrees vs. 6 degrees; *p* = 1).

#### Shape of first metatarsal head

Seventeen cases presented a round shape, compared to the seven cases with a flat or chevron type head of the first metatarsal. There was no significant difference between the two groups (Table 2d). The cases with a round metatarsal head yielded a greater but insignificant progression of the HVA compared to the episodes with a flat or chevron type head (7.6 degrees vs. − 1.7 degrees; *p* = .11) and of the HVI (2.8 degrees vs. 2.2 degrees; *p* = .41). However, they did not show a higher progression of the remaining IMA (− 0.4 degrees vs. 2.2 degrees; *p* = .17).

#### Level of amputation

Fifteen cases underwent amputation through the MTP, compared to nine with transmetatarsal amputation. A supporting effect of a totally preserved second metatarsal bone could not be shown (Table 2e). Cases with transmetatarsal amputation showed a higher progression of the IMA (1.5 degrees vs. − 0.03 degrees; *p* = .6) compared to those with preservation of the metatarsal bone. Conversely, patients with transmetatarsal amputation revealed a lower progression of the HVA (4.3 degrees vs. 5.2 degrees; *p* = .73) and of the HVI (2.2 degrees vs. 2.9 degrees; *p* = .47).

### Interobserver reliability

We could show a very high interobserver reliability between all radiographic measurements between the two orthopedic surgeons with Pearson correlation coefficients between 0.978 (*p* = .01) and 0.999 (*p* = .01).

## Discussion

The primary finding of this study is that 50% of patients undergoing second toe amputation develop ulcers in the contact area of the first and third toe, on average one and a half year after amputation. According to our observations, ulcer localization was similarly distributed between the first and third toe, with a slight preference of the third (42%) over the first (8%) toe. 33% had kissing ulcers. The slight dominance of the third toe might be explained by thinner soft tissue envelopment.

All radiographic parameters for hallux valgus deformity increased, yet not significantly, on average after amputation of the second toe. It has already been described in 1965 that loss of the buttressing effect of the second toe due to amputation might induce lateral drifting of the first toe into the formerly occupied position by the second toe [15]. Similarly, another radiologic investigation showed a correlation of a lateral deviation of the second toe and displacement with an increased hallux valgus deformity of the first toe. However, it was not possible to investigate which deformity occurred first [24]. Other authors observed the deviation of adjacent toes in case of minor amputations as those toes occupy the space that was previously taken by the amputated toe [25]. Finally, a single case report documented the development of a severe hallux valgus deformity in a female patient 2 years after amputation of the second toe due to osteomyelitis and diabetic foot syndrome [26].

Our results did not show a significant correlation between progression of hallux valgus deformity and interdigital ulcerations on the adjacent toes. Other authors considered just the preexistence of this deformity and did not evaluate the postoperative development and progression of specific radiologic hallux valgus parameters. A study with almost 3000 diabetic feet showed that a hallux valgus deformity at baseline was not predictive for development of ulcerations in the future [8]. Another investigation from the same institution with 398 subjects with diabetes mellitus did not find a significant correlation between a special foot or toe type at baseline and ulcer outcome [18]. Similarly, our results did not show a correlation between preexisting hallux valgus deformity and ulcer development.

Further, there was no correlation between BMI and interdigital ulcer development in our cohort. Whereas the literature agrees that patients with a higher BMI are not prone to develop a hallux valgus deformity [23], there is no consensus about the relation between weight and ulcerations. A study with over 100,000 diabetic patients revealed a J-shape association between the risk to develop foot ulcers and the body weight. Thereby, the ulceration risk almost doubles with a BMI > 40, possibly due to the increased ground contact in patients with a higher BMI and subsequent continuous pressure on the foot in walking, but is also higher in patients with normal weight compared to overweight or Class I obese individuals [27]. However, a systematic review that investigated predictive factors for diabetic foot ulcerations, named just 4 out of 25 studies that showed a significant association between a higher BMI and the development of ulcerations [21].

This is the first study investigating interdigital ulcer development due to different metatarsal head shapes. Our results do not indicate that a round shape favors ulcer development and progression of hallux valgus deformity. The literature documents various types of metatarsal heads, ranging from round over chevron type to flat forms, all of them with different biomechanical properties [22, 23]. Thereby, a retrospective study revealed that a round shape of the first metatarsal head is associated with the development of a hallux valgus deformity, possibly due to the negative influence of the head on the intrinsic stability [22]. On the other side, patients with hallux rigidus tend to have a chevron type or flat head of the first metatarsal bone, which may provide more stability against subluxation and deviation to deformity [7].

This is equally the first study analyzing the influence of second metatarsal bone preservation. Although a preserved second metatarsal bone is supposed to provide an important stabilizing effect on the adjacent toes [25], our results did not show significantly more ulcerations or a greater progression of hallux valgus deformity in patients with partial amputation of the second metatarsal bone.

Our study confirms that interdigital ulcers are frequent after amputation of the second toe. For the clinical practice we draw the following conclusions:

1. After second toe amputation, frequent clinical controls are needed to detect clinical signs of pressure on the adjacent first and third toes due to the high rate of interdigital ulcers. As ulcer development occurred after a mean of 20 months, those frequent controls must be upheld at least for 2.5 years. The authors recommend controls every six weeks splitting them evenly between the treating surgeon and an affiliated podiatry service or wound nursery service, if available.
2. Evidence on influencing factors for hallux valgus development and subsequent pressure ulcers after amputation of the second toe is still lacking and neither the BMI, nor the shape of the first metatarsal head or the amputation level seem to be risk factors. Surgeons might consider prescribing a silicone spacer or instruct their patients to use lambswool as a placeholder after amputation of the second toe but there is still no clear evidence which patients benefit from it. Whereas some authors recommend using latex supporting devices for the amputated toe, others prefer total shoe inlays of plastic or lambswool [9].

### Limitation

Besides its retrospective design, our study has the essential limitation of being underpowered. To obtain an adequate power of 0.80 with the probability of a type-I-error of 0.05, 186 participants would be required for this investigation (STATA 9.0, StataCorp LLC, College Station, Texas, USA). This investigation should be the first phase of a following prospective study to establish an algorithm for the management of the amputation of the second toe to investigate advantages of either implantation of a prosthetic device for prevention of lateral drifting of the hallux or prophylactic hallux valgus deformity correction.

## Conclusion

When amputation of the second toe is performed, 50% of the patients develop adjacent toe ulcers within 20 months. Conventional radiographic hallux valgus angles increase as the first toe occupies the space of the missing second toe. However, there are no clear underlying factors that influence its occurrence.

## Data Availability

The analyzed dataset from this investigation is available from the corresponding author on reasonable request.

## Abbreviations

BMI: Body mass index
DMAA: Distal metatarsal articular angle
HVA: Hallux valgus angle
HVI: Hallux valgus interphalangeal angle
IMA: Intermetatarsal angle
